# D-allose suppresses colitis associated carcinogenesis by reversing ER stress in macrophages and inhibiting cancer cell proliferation

**DOI:** 10.3389/fimmu.2026.1737504

**Published:** 2026-02-20

**Authors:** Xiaodong Li, Keizo Hiraishi, Kensuke Kumamoto, Shin-ichi Nakakita, Tetsuo Yamashita, Kazuyo Kamitori, Kiyomi Ohmichi, Ryou Ishikawa, Lin Hai Kurahara

**Affiliations:** 1 Department of Cardiovascular Physiology, Faculty of Medicine, Kagawa University, Kagawa, Japan; 2 Department of Physiology, Faculty of Medicine, Fukuoka University, Fukuoka, Japan; 3 Genome Medical Science and Medical Genetics, Faculty of Medicine, Kagawa University, Kagawa, Japan; 4 Department of Basic Life Science, Kagawa University, Kagawa, Japan; 5 Department of Molecular Physiology, Faculty of Medicine, Kagawa University, Kagawa, Japan; 6 Department of Diagnostic Pathology, Kagawa University Hospital, Kagawa University, Kagawa, Japan

**Keywords:** carcinogenesis, colitis, D-allose, ER stress, macrophages, mitochondrial dysfunction

## Abstract

**Introduction:**

Inflammatory bowel diseases (IBD), especially ulcerative colitis, are associated with a high risk of carcinogenesis. D-allose, a D-glucose epimer, exhibits antioxidant and antitumor activities. This study aimed to examine the effects of D-allose on colitis-associated carcinogenesis.

**Methods:**

A mouse model of colitis-associated carcinogenesis was established followed by treatment with D-allose. *In vitro*, ER stress and mitochondrial function in RAW 264.7 macrophages and the migration and proliferation of Caco-2 cells were analyzed to elucidate the underlying mechanisms. Colonic tissues obtained from IBD patients with were subjected to analyze ER stress in macrophages.

**Results:**

D-allose administration significantly reduced the tumor number, hemorrhage, inflammation score, and macrophage infiltration in the AOM/DSS model. D-allose suppressed ER stress signal and mitochondrial dysfunction in LPS treated RAW 264.7 macrophages. D-allose suppressed ER stress marker Bip and CHOP expression in thapsigargin treated RAW 264.7. In IBD patient’s colon, ER stress marker Bip and CHOP positive macrophage infiltration was detected in both inflammatory and tumor areas. The level of fluorescence labeled M6~G1M9 oligosaccharides increased in the LPS-treated RAW 264.7 macrophages, while thapsigargin or D-allose had no effect. In Caco-2 cells, D-allose suppressed phosphorylated AMPK expression, reduced migratory activity. D-allose inhibited glycolysis, and decreased cell proliferation through TXNIP upregulation.

**Conclusion:**

D-allose suppressed inflammation and tumor development in a colitis-associated carcinogenesis model. D-allose restoring macrophage ER stress and mitochondrial dysfunction, and inhibiting colon cancer cell migration and proliferation. Therefore, D-allose may represent as a promising therapeutic and preventive agent for IBD and inflammation-associated carcinogenesis.

## 1 Introduction

For Inflammatory bowel disease (IBD), including Crohn’s disease (CD) and ulcerative colitis (UC), represents a substantial global health burden owing to the rising number of affected patients (1). Current therapeutic options often fail to achieve sustained remission and may be associated with significant side effects (2). An important concern in IBD management is the increased risk of colitis-associated colorectal cancer (CAC), underscoring the need for novel preventive and therapeutic strategies (3).

Macrophages play a central role in the pathogenesis of IBD and CAC by regulating immune responses and contributing to carcinogenesis (4, 5). Lipopolysaccharide (LPS) activates Toll-like receptor 4 in macrophages, thereby inducing inflammatory responses and promoting the production of proinflammatory cytokines. In addition, LPS stimulates excessive reactive oxygen species (ROS) production through mitochondrial dysfunction, further exacerbating inflammation and oxidative stress (5, 6). A reduction in mitochondrial membrane potential has been associated with excessive ROS production, leading to oxidative damage and the promotion of a proinflammatory macrophage phenotype (7, 8). Endoplasmic reticulum (ER) stress arises as a consequence of inflammation, and excessive ER stress promotes cytokine production and accelerates tumor genesis (9). Severe ER stress can also activate the unfolded protein response, which may promote cell survival or induce mitochondrial-mediated cell death depending on the stress level. Recent studies have suggested that the regulation of ER stress and mitochondrial function contributes to the reduction of inflammation and inhibition of cancer cell proliferation (10). Accordingly, targeting macrophage ER stress regulates IBD pathogenesis and shows promise as a therapeutic strategy for CAC (11, 12).

D-allose, a rare naturally occurring monosaccharide, has attracted attention owing to its antioxidant and anticancer properties (13, 14). Its immunomodulatory effects on cytokine production by plasmacytoid dendritic cells have been previously reported (15). However, the regulation of macrophage function by D-allose has not yet been described. D-allose has demonstrated antioxidant effects activity and inhibits the proliferation and metastasis of various cancer types, including ovarian, prostate, head and neck, leukemia, cervical, and skin cancers (16–21). The inhibition of cancer cell proliferation by D-allose occurs through the specific upregulation of Thioredoxin-Interacting Protein (TXNIP) and subsequent G1 cell cycle arrest in hepatocellular carcinoma cells via stabilization of p27kip1 (22). Furthermore, D-allose suppresses cell growth by decreasing glycolysis and intracellular ATP levels while prolonging adenosine monophosphate-activated protein kinase (AMPK) activation (23). AMPK plays a dual role in regulating cellular migration and invasiveness through the activation of myosin light chain (MLC) phosphorylation (24).

Despite growing evidence of D-allose’s anti-cancer effects, its role in macrophage-mediated inflammation and intestinal carcinogenesis has not been elucidated. In this study, a comprehensive transcriptome analysis was conducted, with a particular focus on macrophage ER stress and mitochondrial function. This study is the first to investigate ER stress in macrophages in the tissues from IBD patients and AOM/DSS model mice. LPS-induced ER stress in macrophages may play an important role leading to immune responses and inflammatory carcinogenesis. The findings suggest that rare sugars may offer a novel therapeutic strategy for the treatment of IBD and colitis-associated cancer.

## 2 Materials and methods

### 2.1 Animal care

The experimental protocol was approved by the Kagawa University Institutional Animal Care and Use Committee and was performed in compliance with the guidelines for conducting animal experiments at Kagawa University (approval number: 19652).

### 2.2 Chemicals and solutions

Azoxymethane (AOM, A5486; Sigma-Aldrich Co., St. Louis, MO, USA) and dextran sulfate sodium (DSS; M.W. 36,000–50,000; CAS number: 9011-18-1; MP Biomedicals, Inc., Santa Ana, CA, USA) were used to establish a mouse model of colitis-associated carcinogenesis. D-allose was provided by the Kagawa University Rare Sugar Research Center and Matsutani Chemical Industry Co. Ltd. D-glucose (SKR 0994) and fluorescein-conjugated phalloidin (068-06261) were purchased from FUJIFILM (Tokyo, Japan). The antibodies used are listed in **Table 1**. Phosphate-buffered saline (PBS) was composed of 8 mM Na_2_HPO_4_·12H_2_O, 2.1 mM NaH_2_PO_4_·2H_2_O, and 140 mM NaCl.

**Table 1:** Antibody list.

Antibody	Dilution	Manufacture	Catalog number	Purpose
β-Actin	1:3000	FUJIFILM	010-27841	WB
Bip	1:1000	Cell signaling	#3177	IH, WB
CD68	1:1000	Cell signaling	#76437	IH
CD206	1:1000	Abcam	Ab64693	IH
CHOP	1:1000	Cell signaling	#2895	IH, WB
F4/80	1:1000	Cell signaling	#70076	IH
Mouse IgG Alexa Fluor 488	1:1000	Cell signaling	#4408s	IH
Mouse IgG Alexa Fluor 546	1:1000	Thermo Fisher	A11018	IH
Mouse IgG HRP	1:3000	Sigama-Aldrich	A4416	WB
Phospho-AMPKα (p-AMPK) (Thr172)	1:1000	Cell signaling	#2535	WB
pMLC2 (Ser19)	1:1000	Cell signaling	#3675	IH WB
ppMLC2 (Thr18/Ser20)	1:1000	Cell signaling	#4370	IH WB
Rabbit IgG Alexa Fluor 488	1:1000	Cell signaling	#4412s	IH
Rabbit IgG Alexa Fluor 546	1:1000	Thermo Fisher	A11071	IH
Rabbit IgG HRP	1:3000	Sigama-Aldrich	A6154	WB

### 2.3 Animal model and experimental conditions

**Figure 1:**
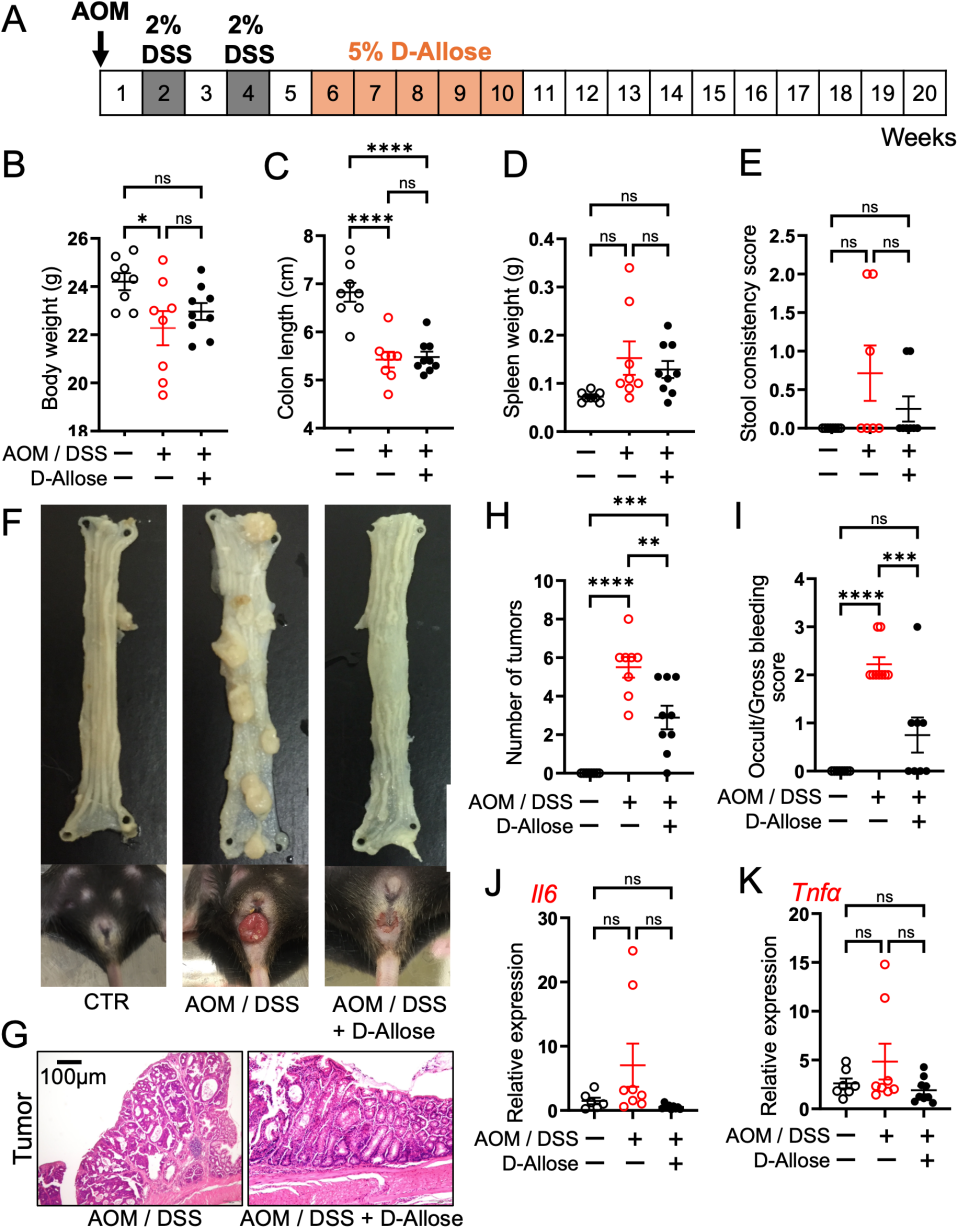
Effects of D-allose on the carcinogenesis in the AOM/DSS mouse model. **(A)** Experimental protocol. **(B–E)** Summary of body weight, colon length, spleen weight, and stool consistency scores at week 20. (n = 8). **(F)** Representative images of the colon and anal areas in each group at week 20. **(G)** Representative H&E-stained image of the mouse colon tumor areas. **(H, I)** Summary of tumor numbers and stool consistency scores (n = 8). **(J, K)** mRNA expression of interleukin 6 and tumor necrosis factor α in the non-tumor colon tissue (n = 6). ns p>0.05, *p < 0.05, **p < 0.01, ***p < 0.001, ****p < 0.0001.

The protocol for animal experiments is illustrated in **Figure 1A**. Eight-week-old female C57BL/6NCrSlc mice (SLC Inc.) were used. The mice were acclimated for more than one week under a 12-h dark/light cycle at a room temperature of 25 °C, with ad libitum access to food and water, until each mouse weighed >20 g. The mice were then randomly assigned to three experimental groups (10 mice per group): control, AOM/DSS, and D-allose treatment (AOM/DSS + D-allose). The control group received intraperitoneal saline and was maintained on standard chow and drinking water. The AOM/DSS group was intraperitoneally administered AOM (12 mg/kg body weight) on day 0 and received standard chow and drinking water. During the second and fourth weeks, 2% DSS was added to the drinking water. The D-allose treatment group followed the same protocol as the AOM/DSS group, with the addition of 5% D-allose in the drinking water during inflammatory tumor genesis phase, from weeks 6 to 10 (**Figure 1A**). 5% D-allose provided consistent modulation of disease index changes without signs of toxicity. Mice were euthanized by cervical dislocation, and spleens were extracted and weighed. The colon segment between the cecum and the anus was excised, and its length was measured. The intestine was then opened longitudinally, and the number of polyps was counted. Sections of the colon, specifically from tumor and non-tumor areas, were collected for downstream analysis.

### 2.4 Histological analysis

Colon tissues from mice were fixed in 10% neutral-buffered formalin and embedded in paraffin. Human colon specimens were obtained from inflamed areas of patients with CD during colonoscopy after obtaining written consent and approval from the Fukuoka University Hospital Ethics Committee (approval number: 12-9-11) (25). After deparaffinization and rehydration, 3-μm-thick tissue sections were subjected to hematoxylin and eosin (HE), Masson’s trichrome (MT), and immunohistochemical staining. The degree of inflammation and fibrosis in mouse colon tissues was evaluated according to the criteria summarized in **Tables 2** and **3**. Scoring was performed by a blinded pathologist at ×200 magnification using HE and MT staining, respectively.

**Table 2:** histology score criteria.

Item	Score	Score				
	Name	0	1	2	3	4
Inflammation	I	none	mild	moderate	severe	
Extend	E	none	mucosa and submucosa	transmural		
Regeneration	R	complete regeneration	almost complete regeneration	regeneration with crypt deletion	surface epithelium not intact	no tissue repair
Crypt damage	C	none	1/3 of basal damaged	2/3 of basal damaged	only surface epithelium intact	entire crypt and epithelium lost
Percent involvement	P	none	1-25%	26-50%	51-75%	76-100%

The total histological score was calculated as I+E+R+C+P.

**Table 3:** Fibrosis score criteria.

Score		
1	mild fibrosis	focal mucosal/submucosal collagen deposition without architectural distortion
2	moderate fibrosis	significant mucosal/submucosal collagen deposition with modest distortion of the mucosal/submucosal architecture but without obscuring the mucosal/submucosal border
3	severe fibrosis	extensive mucosal/submucosal collagen deposition with marked architectural distortion obscuring the mucosal/submucosal border

### 2.5 Immunohistochemistry and immunofluorescence staining

Formalin-fixed, paraffin-embedded specimens obtained from the mice and patients with UC (**Table 4**) were deparaffinized and rehydrated. Antigen retrieval was performed using citric acid (pH 6) at 100°C for 20 min. The sections were then blocked with Blocking One Histo solution (Nacalai Tesque, Kyoto, Japan) or a mouse-on-mouse blocking reagent (Vector Laboratories, Newark, CA, USA) for 10 min at room temperature, followed by incubation with primary antibodies overnight at 4°C. Subsequently, the specimens were incubated with secondary antibodies for 1 hour at room temperature, followed by nuclear counterstaining with TO-PRO-3 iodide (642/661) or 4’,6-diamidino-2-phenylindole (DAPI) (Invitrogen, Thermo Fisher Scientific).

**Table 4:** Clinical IBD sample list.

Number	Tissue	Sex	Age	IBD type
19052	Colon	male	69	UC
19019	Colon	male	56	UC
20027	Colon	male	31	UC
22019	Colon	male	46	UC
22046	Colon	male	52	UC

For immunofluorescence analysis in cultured cells, cells were seeded in 35-mm dishes containing glass coverslips and treated with 50 mM D-allose for 24 hours. The cells were fixed with 4% formalin for 10 min, washed three times with PBS (5 min each), permeabilized 0.1% Triton X-100, and blocked with 10% bovine serum albumin in PBS for 15 min. Incubation with primary antibodies was performed overnight at 4 °C, followed incubation with fluorophore-conjugated secondary antibodies (e.g., anti-mouse Alexa Fluor 488, anti-rabbit Alexa Fluor 555) for 1 hour at room temperature in the dark. After each antibody incubation, the specimens were washed three times with PBS for 5 min. Nuclear staining was performed using 1 μM TO-PRO-3 or DAPI for 15 min, followed by three PBS washes (5 min each). Samples were mounted on glass slides using Fluoromount/PlusTM.

### 2.6 Reverse transcription-polymerase chain reaction analysis

Total RNA was purified using the Tissue Total RNA Mini Kit (Favorgen Biotech Corp., Taiwan, China). A quantity of 500–1,000 μg of total RNA was reverse transcribed with PrimeScript RT Master Mix (Takara Bio Inc., Shiga, Japan). The resulting cDNA (1 μL) was amplified by quantitative polymerase chain reaction (PCR) in a 9-μL reaction mixture prepared with TaqMan Fast Advanced Master Mix (Applied Biosystems, Foster City, CA, USA) and a MicroAmp Fast 96-well Reaction Plate (Applied Biosystems). The primers and probes used are listed in **Table 5**.

**Table 5:** Primer probe list.

Gene name	Vendor	Prime time qPCR assay No.
ACTB	IDT	Mm.PT.39a.22214843.g
il6	Thermo Fisher	Mm00446190_m1
il1β	IDT	Mm.PT.58.8651372
NOS2	IDT	Mm.PT.58.43705194
Tnf	Thermo Fisher	Mm00443258_m1

### 2.7 Cell culture

The human colon carcinoma cell line Caco-2 and the mouse macrophage cell line RAW 264.7 were purchased from the RIKEN BRC (RCB-0988). The cells were maintained in low-glucose Dulbecco’s modified Eagle medium (DMEM; Sigma) supplemented with 1% nonessential amino acids, 1% penicillin/streptomycin, and 10% fetal bovine serum (FBS; Biological Industries, Kibbutz Beit HaEmek, Israel). Cultures were incubated at 37 °C in a humidified atmosphere containing 5% CO_2_.

### 2.8 RNA sequencing analysis of RAW264.7

Total RNA was extracted from RAW264.7 cells using the RNeasy Mini Kit (Qiagen, Hilden, Germany) in accordance with the manufacturer’s instructions. Strand-specific libraries were prepared using the TruSeq^®^ Stranded Total RNA Sample Preparation Kit (Illumina, San Diego, CA, USA) following the manufacturer’s protocol. RNA integrity was evaluated using the Bioanalyzer 2100 system (Agilent Technologies, Santa Clara, CA, USA). Following quality control, libraries were pooled according to effective concentration and the targeted sequencing depth. The 5’-ends of the libraries were phosphorylated and cyclized. Subsequently, loop amplification was performed to generate DNA nanoballs. These nanoballs were loaded onto a flow cell and sequenced using the DNBSEQ-T7 platform. Raw sequencing data in FASTQ format were processed using fastp. During this step, clean reads were generated by removing those containing adapters, poly-N sequences, or low-quality bases. Simultaneously, the Q20, Q30, and GC content values of the clean reads were calculated.

All downstream analyses were performed using clean, high-quality data. The reference genome and gene model annotation files were obtained from the Genome database. An index of the reference genome was constructed using Hisat2 v2.0.5, and paired-end clean 1 reads were aligned to the reference genome. Hisat2 was selected as the mapping tool, as it enables the generation of splice junction databases from gene model annotations, thereby providing superior mapping accuracy compared with non-splice mapping tools. Fold changes were calculated based on the number of fragments per kilobase of exon per million mapped reads for each sample. Differentially expressed genes (DEGs) were defined using a false discovery rate of ≤0.05. To predict the biological functions and pathways associated with DEGs, Gene Ontology (GO) enrichment and Reactome pathway analyses were performed.

### 2.9 Immunoblot analysis

Cell lysates were prepared in radioimmunoprecipitation assay buffer (50 mM Tris-HCl, pH 7.6, 150 mM NaCl, 1% Nonidet P40, 0.5% sodium deoxycholate, 0.1% SDS) supplemented with a proteinase inhibitor cocktail (Nacalai Tesque, Kyoto, Japan). Protein concentrations were determined using a bicinchoninate protein assay kit according to the manufacturer’s instructions (Nacalai Tesque). The samples were adjusted to a concentration of 0.4 μg protein/μL with 4× Laemmli Sample buffer (BIO-RAD, USA) and heated at 95 °C for 5 min. Protein aliquots (2 μg) were separated by sodium dodecyl-sulfate polyacrylamide gel electrophoresis on 8% or 12% polyacrylamide gels and subsequently transferred onto polyvinylidene difluoride (PVDF) membrane (0.45 μm or 0.2 μm pore size; Sigma-Aldrich, St. Louis, MO, USA) using a Power PacTM HC electroblotting system (BIO-RAD, Berkeley, California, USA). Membranes were blocked with Blocking One solution (Nacalai Tesque) for 30 min at room temperature, followed by incubation with primary antibodies diluted in Can Get Signal Immunoreaction Enhancer Solution 1 (Toyobo, Osaka, Japan) overnight at 4 °C. After washing, membranes were incubated with horseradish peroxidase-conjugated secondary antibodies diluted in Can Get Signal Immunoreaction Enhancer Solution 2 (Toyobo) for 1 hour at room temperature. Immunoreactive proteins were visualized using an enhanced chemiluminescence detection system. Chemiluminescence signals were captured with a lumino-image analyzer LAS4000 (Fujifilm, Tokyo, Japan).

### 2.10 Preparation of free oligosaccharide fraction

Cultured cells (>1.0 × 10^6^ cell) collected via centrifugation were subjected to lyophilization. Freeze-dried cells were subsequently extracted with water, methanol, and chloroform at a ratio of 1:1:1. The mixture was centrifuged at 4,000 × g for 10 min, after which the chloroform phase was discarded. The aqueous phase was further clarified by centrifugation and designated as the free oligosaccharide fraction. This fraction was dissolved in 0.1 mL of water and stored at –80 °C until use.

### 2.11 Preparation of pyridylaminated oligosaccharides

The free oligosaccharide fractions were pyridylaminated according to the method of Hase et al. (26, 27). Lyophilized free oligosaccharide fractions (50 μL) were dissolved in 0. 02 mL of coupling reagent (552 mg of 2-aminopyridine in 0.2 ml of acetic acid) and heated at 90 °C for 60 min in a heating block. Following the coupling reaction, 0.07 mL of freshly prepared reducing reagent (200 mg of dimethylamine-borane in 0.08 mL of acetic acid and 0.050 mL of water) was added, and the mixture was subsequently heated at 80 °C for 35 min. The reaction mixture was then extracted with water-saturated phenol/chloroform solution (28), and the pyridylaminated (PA)-free oligosaccharides were obtained and lyophilized. The PA-free oligosaccharides were further purified using a Sep-PAK cartridge (Sep-PAK Plus C18; Waters, MA, Japan). The PA-free oligosaccharides were dissolved in 0.1 mL of 0.1% acetic acid and loaded onto the cartridge. After washing with 3 mL of 0.1% acetic acid, they were eluted with 3 mL of 30% methanol in 0.1% acetic acid (29).

### 2.12 High-performance liquid chromatography analysis of pyridylaminated free oligosaccharides

Each fraction of PA-free oligosaccharides was analyzed by normal phase high-performance liquid chromatography (HPLC) using a TSK gel Amide-80 column (4.6×100 mm) at a flow rate of 0.6 mL/min. Two eluents were used: eluent A (acetonitrile) and eluent B (0.2% ammonium formate, pH 4.4). The column was equilibrated with 20% eluent B. Following sample injection, the concentration of eluent B was linearly increased to 30% in 3 min and subsequently to 65% in 35 min.

### 2.13 Migration assay (chemotaxis assays)

RAW264.7 cells were seeded at a density of 10,000 cells per well in the upper chamber of a Corning^®^ 6.5 mm Transwell^®^ with 8.0 µm pore polycarbonate membrane insert (Corning, NY, USA) using DMEM supplemented with 10% FBS. After 24 hours, the cells in the upper chamber were serum deprived, and migration was stimulated by adding 500 μL of DMEM with 10% FBS to the lower chamber as a positive control. For chemotaxis assays, LPS with and without D-allose was added to the lower chamber in 500 μL of serum-free DMEM. After 24 hours of incubation, the cells were washed with PBS and fixed in 70% ethanol for 10 min at room temperature. Cells on the upper surface of the transwell membrane were gently removed using cotton buds, whereas migrated cells on the lower surface were stained with 0.1% crystal violet for 15 min. Migrated cells were visualized under a light microscope (KEYENCE BIOREVO BZ-9000) equipped with a 40× objective lens (Nikon, Tokyo, Japan). The number of migrating cells was counted in five randomly selected fields.

### 2.14 Mitochondrial production of reactive oxygen species

RAW 264.7 cells were seeded at a density of 50,000 cells per 35-mm dish and cultured in serum-free DMEM supplemented with 1% nonessential amino acids and 1% penicillin/streptomycin, with and without D-allose, for 24 hours. The cells were then washed once with PBS and incubated with 10 μM MitoBright ROS Deep Red (DOJINDO, Kumamoto, Japan) for 30 min at 37°C in a 5% CO_2_ incubator. Fluorescence images (Ex/Em: 561/640–700 nm) were acquired using a Nikon Confocal Microscope System AX, exported in a TIFF format, and quantified using Image J 1.54 g software.

### 2.15 Mitochondrial membrane potential

RAW 264.7 cells were seeded at a density of 50,000 cells per 35-mm dish and cultured in serum-free DMEM containing 1% nonessential amino acids and 1% penicillin/streptomycin, with and without D-allose, for 24 hours. The cells were then washed once with PBS and incubated with a JC-1 Dye kit (MT09, DOJINDO, Kumamoto, Japan) for 30 min at 37°C in a 5% CO_2_ incubator. Following incubation, the cells were then washed twice with a medium, and the imaging buffer solution was added. Green fluorescence (Ex 488 nm/Em 500–550 nm) and red fluorescence (excitation 561 nm/Em 560–610 nm) were observed under a Nikon Confocal Microscope System AX. Images were exported in a TIFF format, and ImageJ 1.54 g software was used to quantify fluorescence intensities. The ratio of green and red fluorescence was calculated as an indicator of mitochondrial membrane depolarization.

### 2.16 Wound healing assay

Caco-2 cells were seeded at a density of 35,000 cells per well in culture-insert 2 well/μ-Dish 35 mm (ibidi GmbH, Germany). After 24 hours of incubation, when confluence was reached, the culture insert was removed to create a gap, initiating wound healing in serum-free DMEM supplemented with 1% nonessential amino acids and 1% penicillin/streptomycin, with and without D-allose. After 24 hours, cell migration into the wound area was evaluated under an optical microscope (Boirevo BZ-9000; KEYENCE, Osaka, Japan).

### 2.17 Transfection of small interfering RNA

Caco-2 cells were seeded at 60% confluence and cultured for 24 hours prior to transfection. The cells were transfected with 30 nM siRNA using Lipofectamine RNAiMAX (Life Technologies, Carlsbad, CA, USA) according to the manufacturer’s protocol. After 72 hours, the cells were subjected to metabolic or cell viability analyses. Knockdown efficiency was evaluated by reverse transcription-PCR at 24, 48, and 72 hours after transfection. The siRNA sequences are listed in **Table 6**.

**Table 6:** Small interfering RNA sequences.

Target	Sense strand(5’-3’)	Antisense strand(5’-3’)
TXNIP-1①	GCAACAUCCUUAGAGUUG	GAAUAUUCAACUCGAAGG
TXNIP-1②	GGUAAUUGGCAGCAGAU	UAGACCUGAUCUGCUGCC
TXNIP-1③	AAGCAGCUUUACCUACUU	AAGAAACAAGUAGGUAAA

### 2.18 Cell proliferation analysis

Cell proliferation was assessed using Cell Counting Kit-8 (CCK-8) (NE100, DOJINDO, Kumamoto, Japan). Caco-2 cells were seeded at 5,000 cells/well in 96-well plates and incubated for 3 days under concurrent exposure to siRNA, as described above. Within 24 hours after siRNA administration, the cells were treated with 50 mM D-allose or D-glucose for 2 days. After washing twice with PBS, a 10% CCK-8 solution diluted in culture medium was added, and the plates were incubated for 2 hours in a cell incubator. The absorbance was then measured at 450 nm using a spectrophotometer (SH-9000Lab; CORONA Electric, Hitachinaka, Japan).

### 2.19 Cell metabolic analysis

Cell metabolism was analyzed using the Seahorse XF HS Mini Analyzer (Agilent, Santa Clara, CA, USA) following treatment or siRNA transfection, as described above. Caco-2 cells were trypsinized and re-plated at 1 × 10^4^ cells/well on an XF HS Mini plate (Agilent, Santa Clara, CA, USA) in DMEM supplemented with 10% FBS and cultured for 24 hours prior to measurement. The XF Calibrant solution and utility plate containing injector ports and probes were filled with distilled water (200 μL/well) and placed in a CO_2_-free incubator at 37 °C overnight. The XF Calibrant solution was added to the utility plate 2 hours before calibration. After 24 hours of stimulation, the culture medium was replaced with DMEM XF assay medium (pH 7.4; Agilent, Santa Clara, CA, USA) at 180 μL/well. The XF assay medium was supplemented with 10 mM glucose, 1 mM pyruvate, and 2 mM glutamine (Agilent Technologies). The cell culture plates were then placed in a CO_2_-free incubator for 45 min. Oligomycin (ATPase inhibitor, 3 μM), carbonyl cyanide-p-trifluoromethoxyphenylhydrazone (3 μM), and rotenone/antimycin A (0.5 μM) were added to the appropriate ports of the utility plate for a standard Mito Stress test, according to the manufacturer’s instructions (Agilent, 103015-100). For the glycolytic rate assay, rotenone/antimycin A (0.5 μM) and 2-deoxy glucose (50 mM) were sequentially added to the appropriate ports of the utility plate, according to the manufacturer’s protocol. The plate was initially run on a flux analyzer for calibration. The utility plate was then replaced with the cell culture plate and analyzed in real time on the Seahorse XF analyzer using the software’s Mito Stress Test or Glycolytic Rate Assay template program.

### 2.20 Statistical analyses

The value of n indicates the number of independent experiments or animals used. Significant differences were evaluated by analysis of variance, followed by the Tukey–Kramer test for multiple comparisons, using Prism-GraphPad software version 10.3.1 (Subscription ID: 2942871). A p value of <0.05 was considered significant.

## 3 Results

### 3.1 D-allose treatment ameliorated carcinogenesis in the AOM/DSS model

The effect of D-allose on enteritis and carcinogenesis was examined by administering 5% D-allose in drinking water to AOM/DSS mice (**Figure 1A**). The AOM/DSS group exhibited significant reductions in body weight and colon length compared with the control (CTR) group. The AOM/DSS + D-allose group exhibited significant reduction colon length compared with the CTR group (**Figures 1B, C**). No significant differences were observed in spleen weight or stool consistency scores among the three groups (**Figures 1D, E**). Anal tumor prolapse was frequently observed in the AOM/DSS group but not in the CTR or AOM/DSS + D-allose groups (**Figure 1F**). The AOM/DSS group showed increased tumor numbers and higher occult/gross bleeding scores. Histological analysis revealed moderately to well-differentiated adenocarcinomas in both D-allose-treated and untreated AOM/DSS groups (**Figures 1G–I**). Notably, D-allose treatment significantly reduced both tumor numbers and bleeding scores (**Figures 1H, I**). However, the mRNA expression levels of inflammatory cytokines interleukin (IL)-6 and tumor necrosis factor (TNF)-α in the non-tumor colonic area did not differ significantly among the three groups (**Figures 1J, K**).

**Figure 2:**
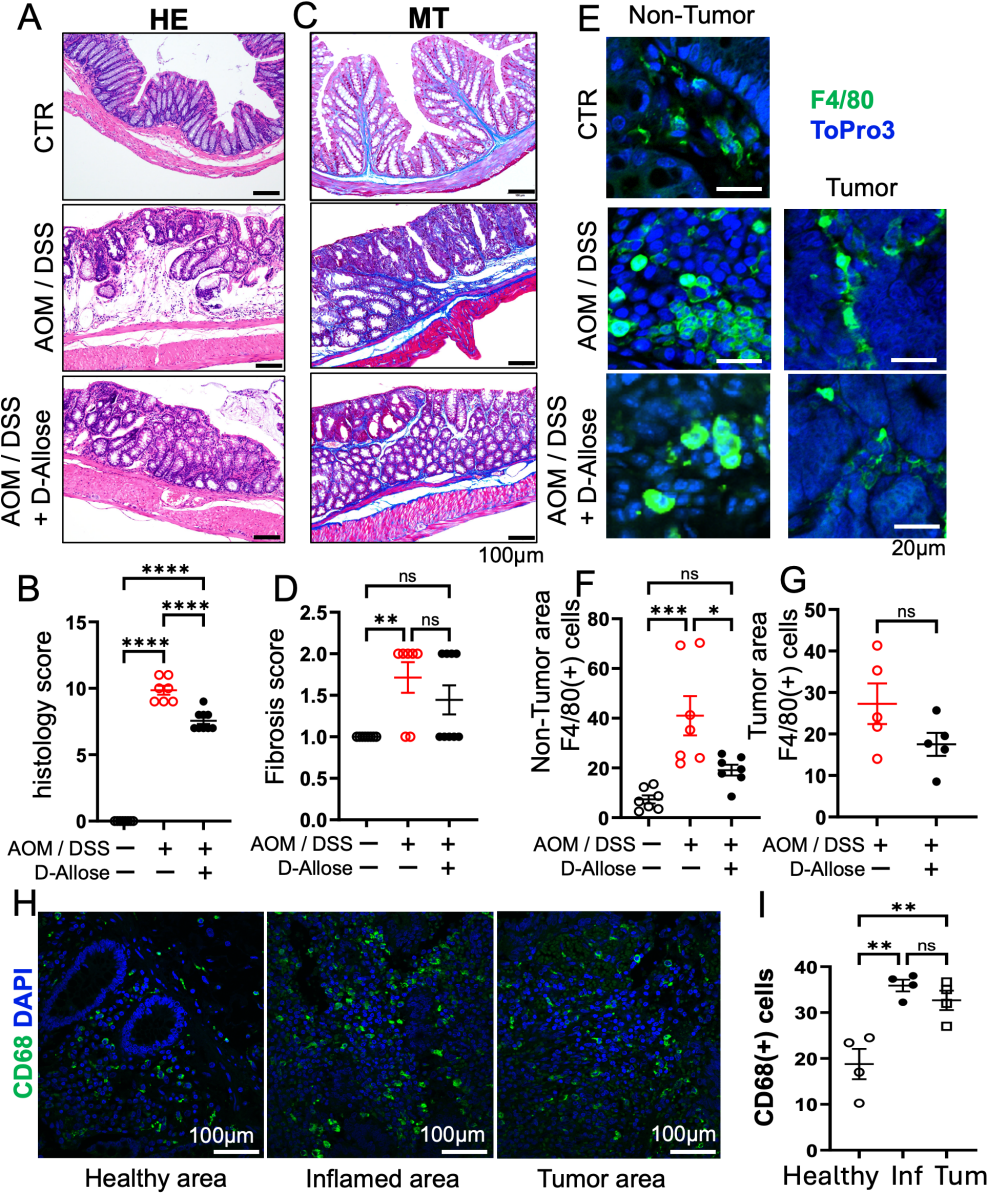
Effects of D-allose on inflammation and fibrosis in the AOM/DSS mouse model. **(A, B)** Representative H&E-stained images of the mouse colon and a summary of inflammation scores (n = 7). **(C, D)** Representative Masson’s trichrome-stained images of the mouse colon and a summary of the fibrosis scores (n = 7). **(E)** Immunohistochemical staining of the mouse colon tissue showing F4/80 expression; nuclei were stained with ToPro3 (blue). **(F, G)** Statistical analysis of the F4/80-positive cells in **(E)**. **(H)** Immunohistochemical staining of human colon tissue showing CD68 expression; nuclear staining with DAPI (blue). **(I)** Statistical analysis of CD68-positive cells in Figures **(H)** ns p > 0.05, * p < 0.05, ** p < 0.01, *** p < 0.001, and **** p < 0.0001.

The AOM/DSS group developed a significantly higher number of colon tumors compared with the control group. By contrast, D-allose treatment significantly reduced tumor numbers (**Figure 1H**). The AOM/DSS group exhibited more pronounced tumorigenesis, stromal fibrosis, and inflammatory cell infiltration compared with the control group (**Figures 2A, B**). D-allose treatment attenuated inflammatory cell infiltration (**Figures 2A, B**) but had no effect on stromal fibrosis (**Figures 2C, D**). In the non-tumor areas, the number of F4/80-positive cells was increased in the AOM/DSS group and significantly reduced by D-allose treatment (**Figures 2E, F**, **Supplementary Figure 1**), and D-allose suppressed the F4/80 and CHOP double positive cells (**Supplementary Figures 2A, B**). No significant difference in the number of F4/80-positive cells was observed in tumor areas between D-allose-treated and untreated groups (**Figures 2E, G**). In addition, we found that CD206 positive type 2 macrophages are decreased in the tumor area by D-allose treatment (**Supplementary Figures 2C, D**). In human colon tissues, CD68-positive cells were more abundant than in healthy CTRs and were similarly distributed between non-tumor and tumor regions (**Figures 2H, I**).

### 3.2 Suppression of macrophage ER stress in RAW 264.7 cells by D-allose

**Figure 3:**
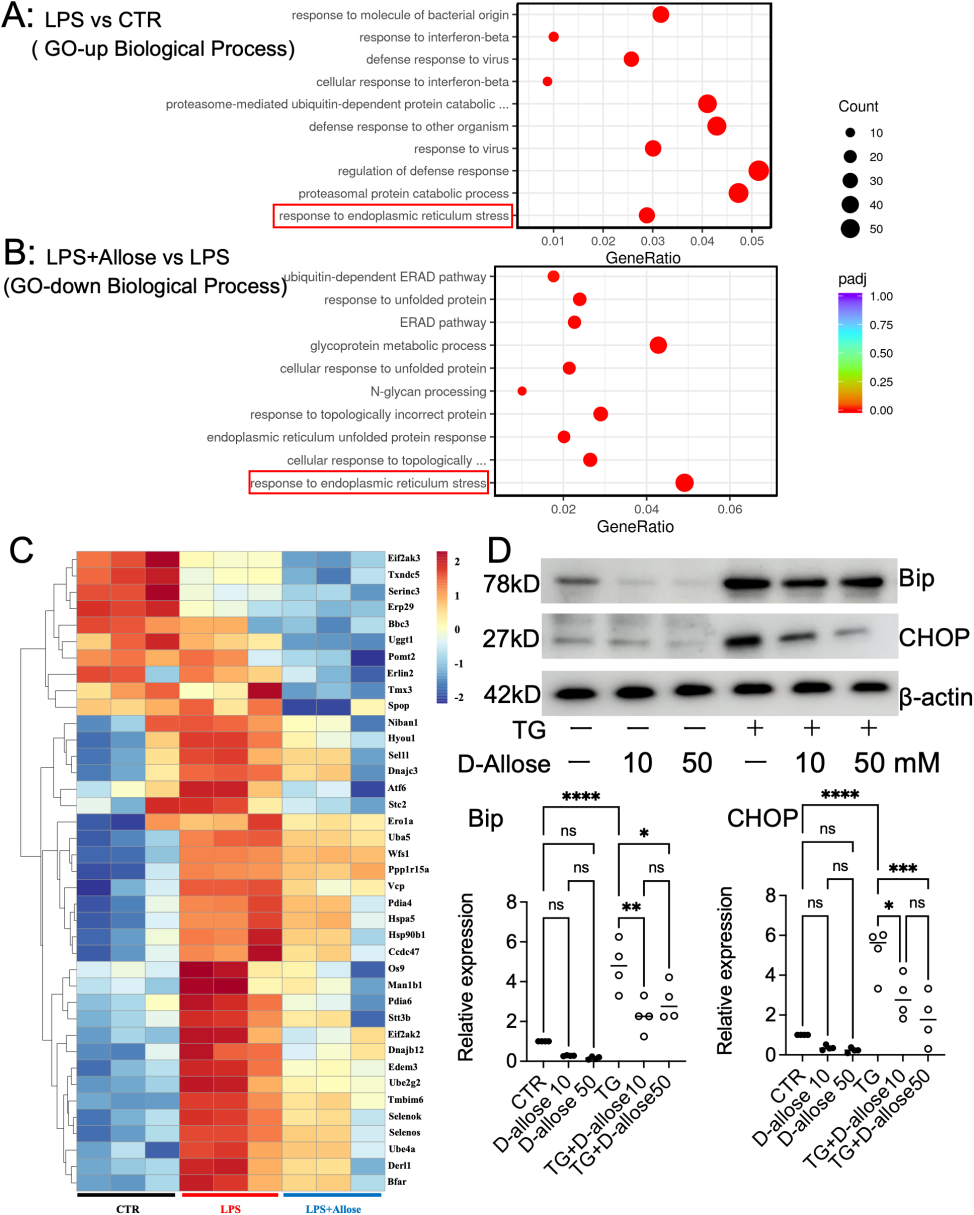
Transcriptome analysis and ER stress evaluation in RAW264.7 cells. **(A)** Upregulated pathways identified by Gene Ontology (GO, Biological Process) analysis of LPS (1 μg/mL, 24 hours) vs. CTR. **(B)** Downregulated pathways identified by GO analysis (Biological Process) of LPS (1 μg/mL, 24 hours) + D-allose (50 mM, 24 hours) vs. LPS (1 μg/mL, 24 hours). **(C)** Heatmap of downregulated genes associated with [response to endoplasmic reticulum stress] following D-allose treatment (n = 3). **(D)** Western blot analysis of Bip and CHOP in RAW264.7 cells treated with or without thapsigargin (0.2 μM) or D-allose (10 or 50 mM) for 24 hours (n = 4) ns p > 0.05, * p < 0.05, ** p < 0.01, *** p < 0.001, and **** p < 0.0001.

A comprehensive transcriptome analysis was conducted to investigate the effects of D-allose on macrophages stimulated with LPS in RAW 264.7 cells. Based on the GO pathway database, genes significantly upregulated by LPS stimulation were prominently associated with ER stress (**Figure 3A**). When the LPS + D-allose group was compared with the LPS group, ER stress-related pathways were significantly downregulated, as illustrated in the heatmap (**Figures 3B, C**). To evaluate ER stress at the protein level, the expression of representative ER stress proteins, binding immunoglobulin protein (Bip) and C/EBP homologous protein (30), was examined by immunoblotting. However, LPS stimulation did not result in increased expression of Bip or CHOP (**Supplementary Figure 3**). Consequently, the effects of D-allose on macrophages stimulated with thapsigargin, a well-established ER stress inducer, were further evaluated. Under these conditions, D-allose suppressed the thapsigargin-induced upregulation of Bip and CHOP expression (**Figure 3D**).

**Figure 4:**
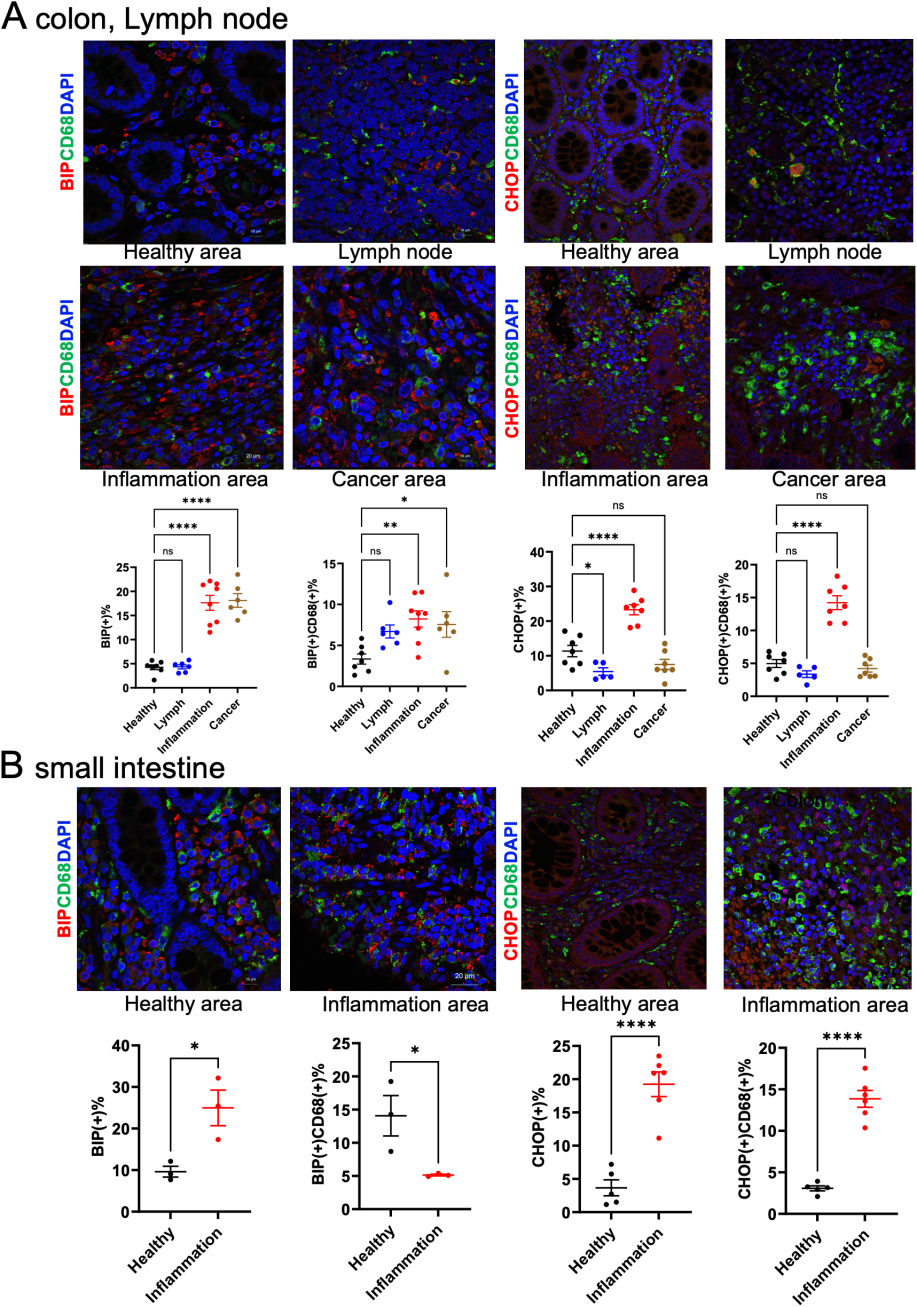
Macrophage ER stress analysis in clinical samples. **(A)** Immunohistochemical staining of colon and lymph node tissues from patients with IBD. **(B)** Immunohistochemical staining of small intestine tissues from IBD patients. CD68 was used as a macrophage marker and was co-stained with Bip or CHOP. Positive cell numbers were normalized to DAPI-positive nuclei (n = 5). ns p>0.05, * p < 0.05, ** p < 0.01, *** p < 0.001, **** p < 0.0001.

In intestinal tissues from patients with CD and UC, the expression of ER stress markers Bip and CHOP was colocalized with macrophage marker proteins (**Figures 4A, B**). The number of macrophages expressing Bip was significantly increased in both inflammatory and carcinogenic regions of the colon, whereas macrophages expressing CHOP were significantly increased in inflammatory regions.

### 3.3 HPLC profiles of pyridylaminated free oligosaccharides

**Figure 5:**
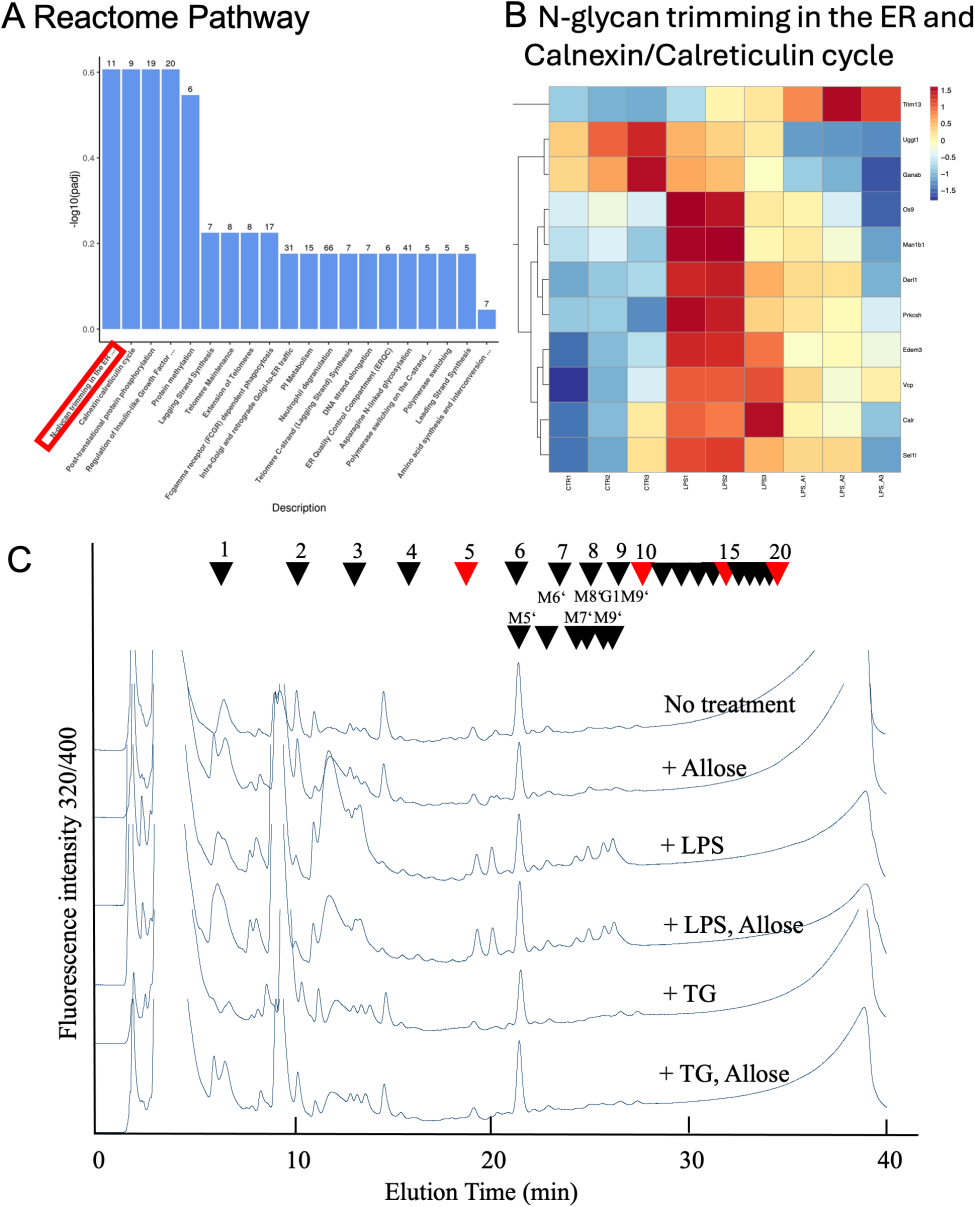
NP-HPLC Profiles of Free oligosaccharides. **(A)** Reactome pathways analysis of RAW264.7 cell transcriptomes treated with LPS (1 μg/mL, 24 hours) + D-allose (50 mM, 24 hours) (n = 3). **(B)** Gene list of N-glycan trimming in the ER and the Calnexin/Calreticulin cycle from Reactome pathway analysis of RAW264.7 cell transcriptomes (n = 3). **(C)** Free oligosaccharides were isolated from RAW264.7 cells treated with or without LPS (1 μg/mL), thapsigargin (0.2 μM), or D-allose (50 mM) for 24 h (n = 4), labeled with fluorescent tags, and separated by NP-HPLC. Arrowheads indicate the elution position of major free oligosaccharide (n = 3).

According to the Reactome pathway database, transcriptome pathway analysis in RAW 264.7 cells indicated that the D-allose treatment significantly changed N-glycan trimming in the ER and Calnexin/Calreticulin cycle (**Figure 5A**). Therefore, the analysis was focused on free oligosaccharides in the RAW 264.7 cells treated with or without D-allose, LPS, and thapsigargin. Free glycans were isolated from drug-treated cells, labeled with fluorescent tags, and separated by HPLC (**Figure 5B**). The arrowheads indicate the elution positions of the major free oligosaccharides. The amount of glycosylated free oligosaccharides was increased in LPS-treated cells compared with the control group, whereas no change was observed in the thapsigargin or D-allose-treated RAW 264.7 cells (**Figure 5C**).

### 3.4 Mitigating effects of D-allose on inflammation, migration, and mitochondrial dysfunction in RAW264.7 cells

**Figure 6:**
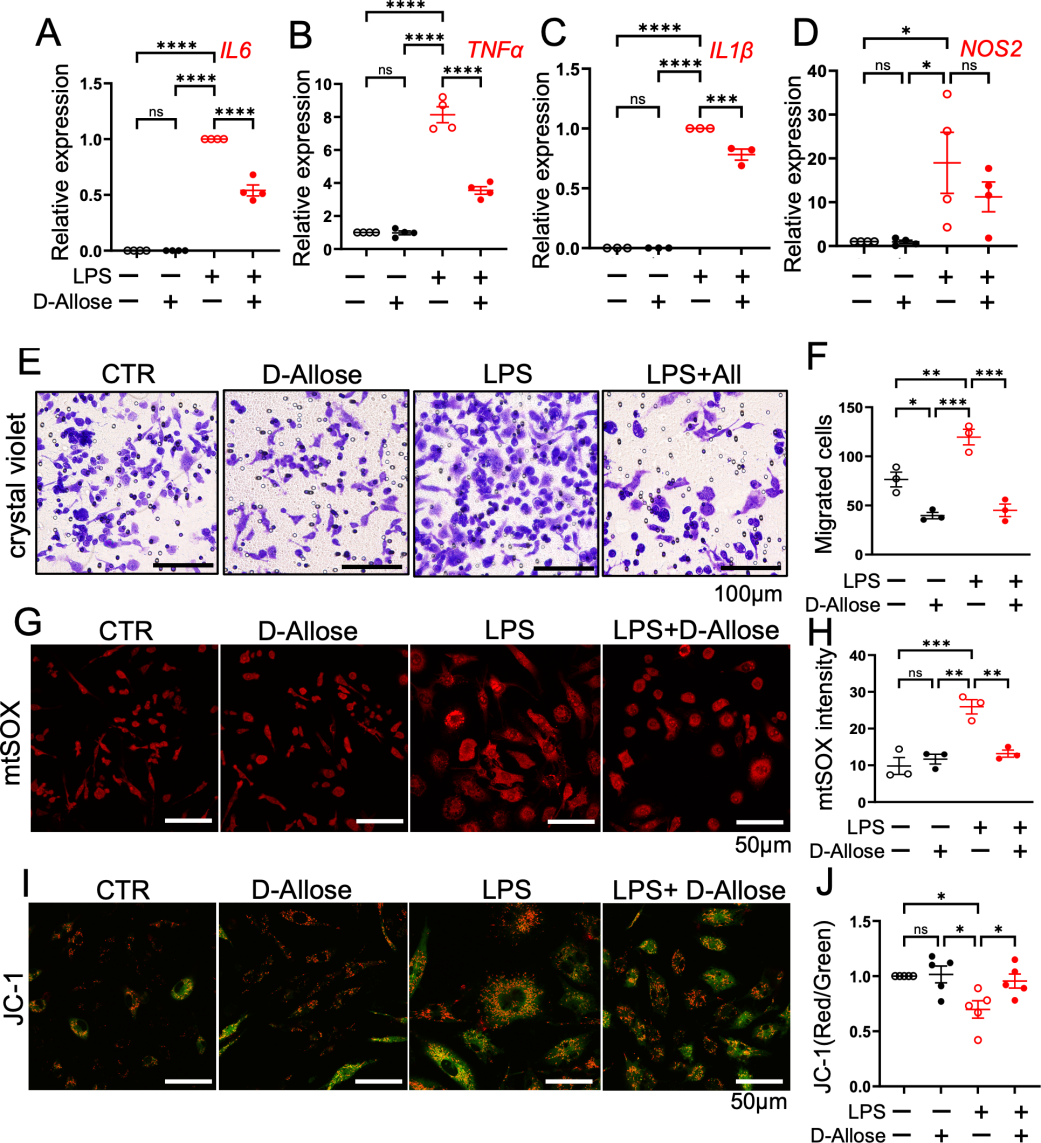
Effects of D-allose on cytokine expression, migration, and mitochondrial function in RAW264.7 cells. RAW264.7 macrophage were treated with D-allose (50 mM), LPS (1 μg/mL), and LPS + D-allose for 24 hours. **(A–D)** Real-time PCR analysis of the inflammatory cytokines IL-6, TNF-α, IL-1β, and NOS2. **(E, F)** Crystal violet staining to assess cell migration. **(G, H)** mtSOX staining to evaluate mitochondrial reactive oxygen species levels. **(I, J)** JC-1 staining red/green ratio indicates the mitochondrial membrane potential. Polarized mitochondria are indicated by red fluorescence (JC-1 dimers), whereas depolarized mitochondria are indicated by green fluorescence (JC-1 monomers). ns p>0.05, * p < 0.05, ** p < 0.01, *** p < 0.001, **** p < 0.0001.

D-allose reduced macrophage infiltration in AOM/DSS-fed mice (**Figure 2E**). To further investigate its effects, an *in vitro* inflammation model was employed using LPS-treated RAW264.7 cells. D-allose markedly reduced the LPS (1 μg/mL, 24 h)-induced upregulation of IL-6, TNFα, and IL-1β expression (**Figures 6A–D**). D-allose inhibited cell migration in both CTR and LPS-treated RAW264.7 cells (**Figures 6E, F**). In addition, D-allose attenuated mitochondrial ROS production induced by LPS treatment (**Figures 6G, H**) and restored the LPS-induced reduction in mitochondrial membrane potential in RAW264.7 cells (**Figures 6I, J**).

### 3.5 Inhibitory effects of D-allose on cell migration and AMPK phosphorylation in Caco-2 cells

**Figure 7:**
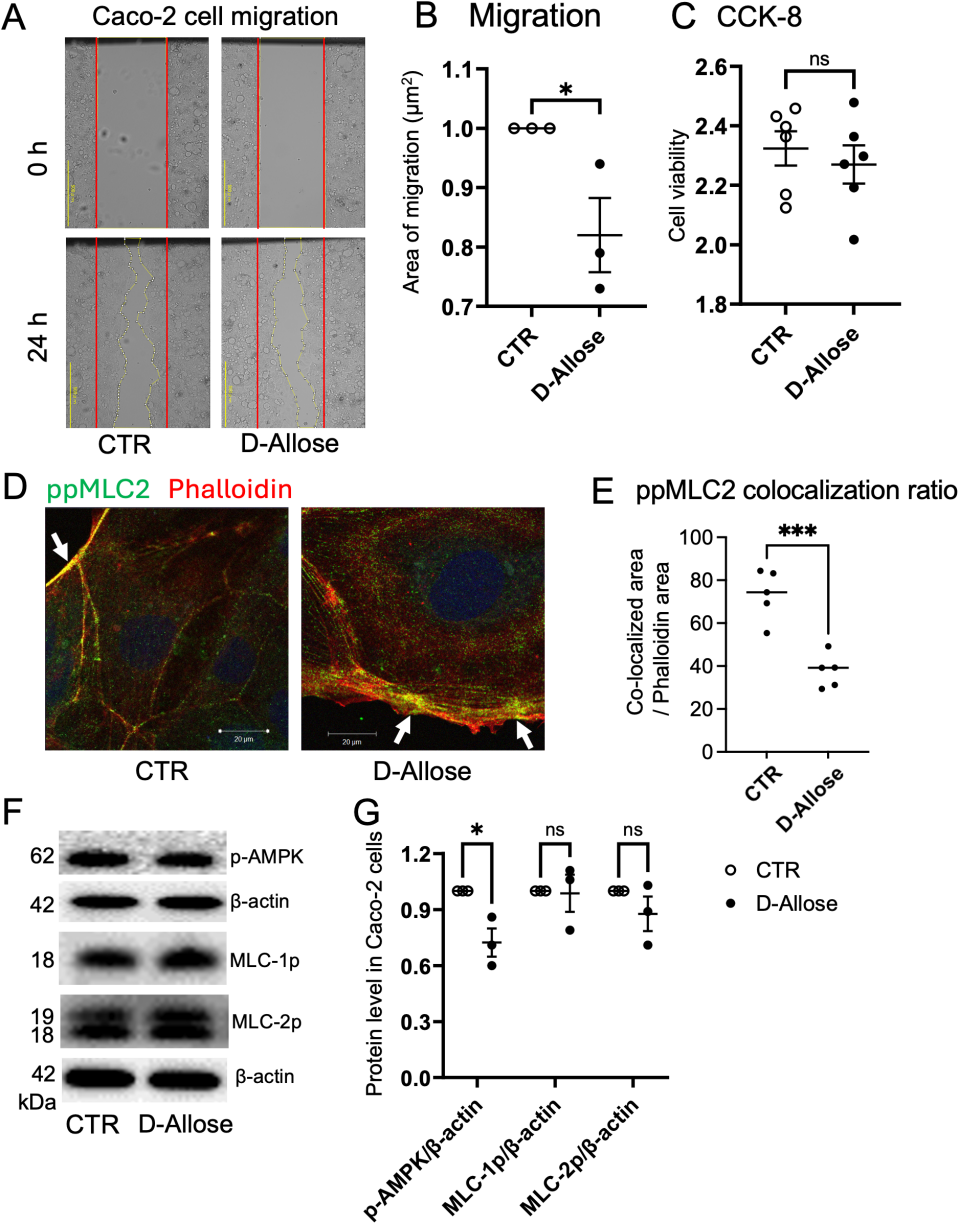
Effect of D-allose on Caco-2 cell migration. **(A, B)** Migration assay of Caco-2 cells treated with or without D-allose (50 mM) for 24 hours. **(C)** Viability of Caco-2 cells assessed using the CCK-8 kit. **(D, E)** Immunofluorescence staining of ppMLC2 and phalloidin (actin: red) in Caco-2 cells treated with or without D-allose (50 mM) for 24 hours. Colocalization ratios of ppMLC2 and phalloidin were quantified. **(F, G)** Western blot analysis of pAMPK, pMLC2, and ppMLC2 levels in Caco-2 cells treated with or without D-allose (50 mM) for 24 hours. ns p>0.05, * p < 0.05, *** p < 0.001.

In the wound healing assay, 24-hours treatment with D-allose (50 mM) decreased the migration of Caco-2 cells without affecting the cell number (**Figures 7A–C**). Immunofluorescence staining further demonstrated reduced colocalization of phosphorylated MLC (ppMLC) and actin in D-allose-treated cells, suggesting that D-allose may attenuate cell contractility and motility through the downregulation of the ppMLC-actin interaction (**Figures 7D, E**). No significant colocalization of pMLC and phalloidin was observed, regardless of D-allose stimulation (**Supplementary Figure 4A**). Western blot analysis revealed a significant decrease in the phosphorylation of AMPK in the D-allose-treated group, whereas no effect was observed on the phosphorylation of pMLC2 and ppMLC2 (**Figures 7F, G**).

### 3.6 Suppression of cell proliferation in a TXNIP-dependent manner in Caco-2 cells by D-allose

The effects of D-allose on the proliferation and metabolism of colon cancer cells were investigated to elucidate the mechanisms underlying its anticancer activity. Caco-2 cells were cultured in low-glucose DMEM, and the effects of D-allose were compared with those of D-glucose at the same concentration.

**Figure 8:**
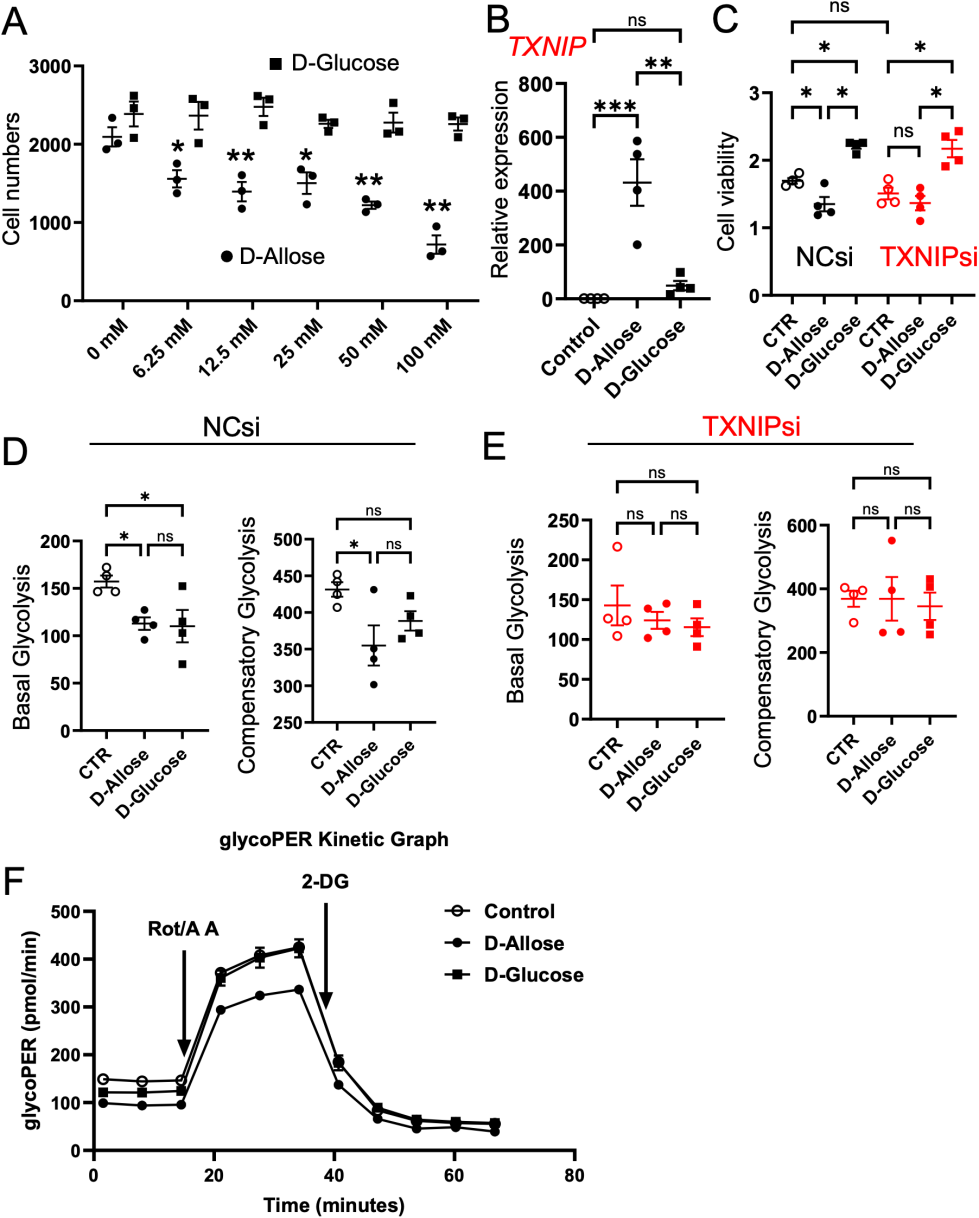
Effects of D-allose on proliferation and glycolysis of Caco-2 cells. **(A)** Cell counts of Caco-2 cells treated with varying concentrations of D-glucose or D-allose for 48 hours (n = 6). **(B)** TXNIP mRNA expression in Caco-2 cells treated with 50 mM D-glucose or D-allose for 72 hours (n = 4). **(C)** Viability of Caco-2 cells transfected with negative control siRNA (NCsi) or TXNIP-targeted siRNA (TXNIPsi) and treated with 50 mM D-allose or D-glucose for 72 hours (n = 6). **(D, E)** Glycolysis assessment in Caco-2 cells transfected with NCsi or TXNIPsi. **(F)** Representative glycoPER kinetics graph of Caco-2 cells treated with 50 mM D-allose or D-glucose for 72 hours (n = 4). ns p>0.05, * p < 0.05, ** p < 0.01, *** p < 0.001.

D-allose significantly reduced the number of viable Caco-2 cells in a concentration-dependent manner, with significant inhibition observed at a concentration of ≥6.25 mM, whereas D-glucose had no effect (**Figure 8A**). D-allose also markedly upregulated the TXNIP mRNA expression, whereas D-glucose produced no effect (**Figure 8B**). To investigate the role of TXNIP in cell proliferation and energy production, TXNIP expression was knocked down using siRNA. Successful knockdown of TXNIP mRNA by three different siRNAs was confirmed 72 hours post-transfection (**Supplementary Figure 4B**). TXNIPsi-3, the most effective siRNA, was selected for subsequent experiments. Following TXNIP knockdown, D-allose failed to suppress cell viability (**Figure 8C**). Meanwhile, D-glucose significantly increased the number of viable Caco-2 cells irrespective of transfection with negative CTR or TXNIP-targeting siRNA (**Figure 8C**). Although TXNIP increased Glucose Transporter 1(GLUT1) mRNA expression, neither D-allose nor D-glucose affected Glut1 mRNA levels in Caco-2 cells (**Supplementary Figure 4C**).

Aerobic glycolysis is a metabolic process that promotes cancer cell proliferation (31). D-allose significantly reduced basal and compensatory glycolysis in Caco-2 cells; however, these inhibitory effects were abolished following TXNIP knockdown (**Figures 8D–F**). D-glucose significantly suppressed basal glycolysis but had no effect on compensatory glycolysis. Its inhibitory effect was similarly abolished by TXNIP knockdown.

The effect of D-allose on mitochondrial respiration in Caco-2 cells was further investigated using the Mito Stress Test kit (Agilent). Neither D-allose nor D-glucose significantly affected basal respiration, maximal respiration, ATP production, or spare respiratory capacity, irrespective of transfection with the negative CTR or TXNIP-targeting siRNA (**Supplementary Figures 5A, B**). Notably, TXNIP siRNA-treated cells exhibited significantly lower basal respiration, maximal respiration, and ATP production compared with negative CTR siRNA-treated cells (**Supplementary Figure 5C**).

## 4 Discussion

This study demonstrates that D-allose exerts both anti-inflammatory and antitumor effects in a mouse model of CAC. *In vitro* experiments further revealed that D-allose suppresses ER stress and mitochondrial dysfunction in macrophages and attenuates colon cancer cell migration and proliferation. These findings highlight the potential of D-allose as a therapeutic candidate for the prevention and treatment of inflammation-associated tumorigenesis.

In an AOM/DSS-induced CAC mouse model, D-allose administration significantly suppressed tumor progression and alleviated rectal bleeding. Notably, D-allose reduced macrophage infiltration in inflamed colonic tissues, consistent with previous findings using the same protocol, and indicating that macrophages are key mediators of inflammation-driven carcinogenesis in IBD (32). Although D-allose exerted limited effects on overall fibrosis or cytokine levels *in vivo*, its selective effect on immune cell dynamics suggests a potential immunomodulatory role, potentially mediated by the gut microbiota, known to metabolize the rare sugars and influence local immune responses (32, 33). Importantly, D-allose mitigated ER stress and mitochondrial dysfunction in macrophages under inflammatory stimulation. In RAW264.7 macrophages, D-allose effectively suppressed LPS-induced expression of inflammatory cytokines (IL-6, TNF-α, and IL-1β), attenuated ROS production, and alleviated mitochondrial dysfunction. These findings suggest that D-allose exerts anti-inflammatory effects by preserving ER and mitochondrial function, suppressing ROS, and modulating multiple interrelated pathways that regulate macrophage behavior in IBD. Transcriptome analysis further demonstrated that D-allose suppressed the expression of N-glycan trimming-related genes, although no changes in free glycans were observed. Additional studies are required to further elucidate the inhibitory effect of D-allose on macrophage ER stress, including the potential involvement of autophagy (34).

In Caco-2 cells, D-allose robustly increased TXNIP expression, resulting in the suppression of glycolysis and the inhibition of cell proliferation and migration. These findings are consistent with previous reports demonstrating that TXNIP functions as a metabolic checkpoint in cancer cells by negatively regulating glucose uptake and glycolysis (14, 35). Although D-allose strongly suppressed glycolysis, mitochondrial respiration was unaffected, suggesting that its effects are specific to glycolytic metabolism. The loss of TXNIP may promote cancer progression by enhancing glycolysis and supporting the Warburg effect, which reflects a preference for aerobic glycolysis over oxidative phosphorylation in cancer cells. In head and neck cancer cell lines, D-allose has been reported to promote AMPK phosphorylation, a key regulator of energy metabolism (23). By contrast, our results demonstrated that D-allose phosphorylated AMPK expression in Caco-2 cells, reduced ppMLC2–actin colocalization, and inhibited colon cancer cell migration. This observation aligns with previous evidence that regulation of MLC2 phosphorylation by phosphorylated AMPK modulates cell migration in cancer cells (24).

Several limitations of this study should be acknowledged. First, the *in vivo* findings were derived from a single AOM/DSS-induced mouse model of colitis-associated cancer, which may not fully capture the heterogeneity of human IBD-associated tumorigenesis. Second, although macrophage infiltration and stress responses were analyzed, macrophage subtype-specific functions and polarization states were not directly interrogated. Third, the therapeutic effect of D-allose could not be observed by quantifying the mRNA levels of inflammatory cytokines in non-tumor area. Future studies should directly quantify inflammatory cytokines in the tissues and blood. Finally, future studies should investigate the involvement of gut microbiota in the anti-inflammatory and carcinogenic effects of D-allose *in vivo*. The AOM/DSS model is a carcinogenesis model that is strongly influenced by gut microbiota, and the *in vitro* macrophage-specific ER stress regulation by D-allose under LPS stimulation also suggests gut microbiota-related inflammatory signals.

Taken together, these results suggest a dual-action mechanism in which D-allose concurrently targets macrophages related immune dysregulation and cancer metabolism in IBD/CAC. Its unique pharmacological profile distinguishes it from current IBD therapies, such as biologics or anti-TNF agents, which primarily suppress inflammation without addressing metabolic dysfunction or tumor risk.

## 5 Conclusion

This study identifies D-allose as a novel immunometabolic modulator that suppresses inflammation and colorectal tumor development. Its effects are mediated through the alleviation of ER stress and restoration of mitochondrial homeostasis in macrophages and reprogramming of cancer cell metabolism via TXNIP. These findings highlight the potential of rare sugars as adjunctive or preventive therapies in IBD and CAC and underscore the therapeutic value of targeting the intersection between metabolism and immunity in chronic inflammatory diseases.

## Data Availability

The RNA sequence data reported in this paper have been deposited in the DNA Data Bank of Japan (DDBJ), BioProject Accession ID: PRJDB39744.
