# Supplementary material for: D-allose suppresses colitis associated carcinogenesis by reversing ER stress in macrophages and inhibiting cancer cell proliferation
Source: Front Immunol. 2026 Feb 20;17:1737504. doi: 10.3389/fimmu.2026.1737504 (PMC12963985; doi:10.3389/fimmu.2026.1737504)

Supplementary Material

# Supplementary Figures

**Supplemental figure 1: Original data of Figure2E**


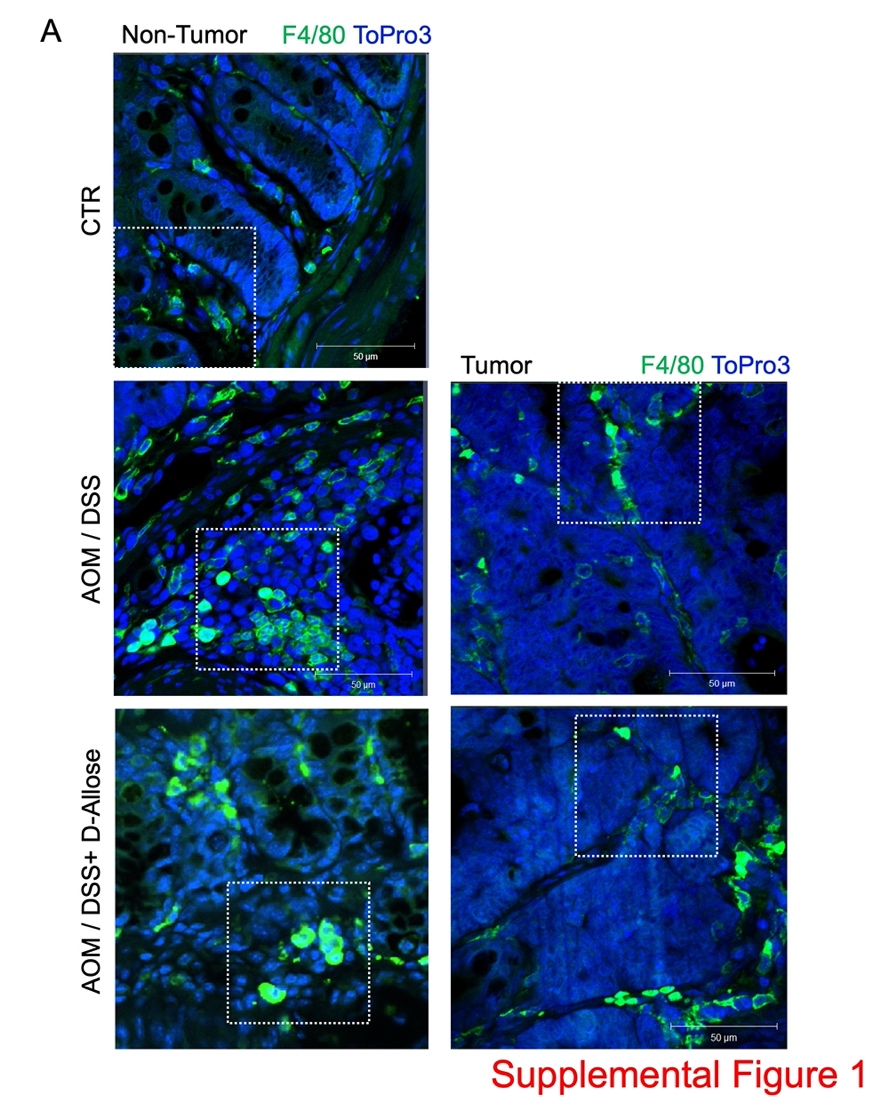


Immunohistochemical staining of the mouse colon tissue showing F4/80 expression; nuclei were stained with ToPro3 (blue).

**Supplemental figure 2: macrophages ER stress in the non-tumor area and type 2 macrophages in tumor area**


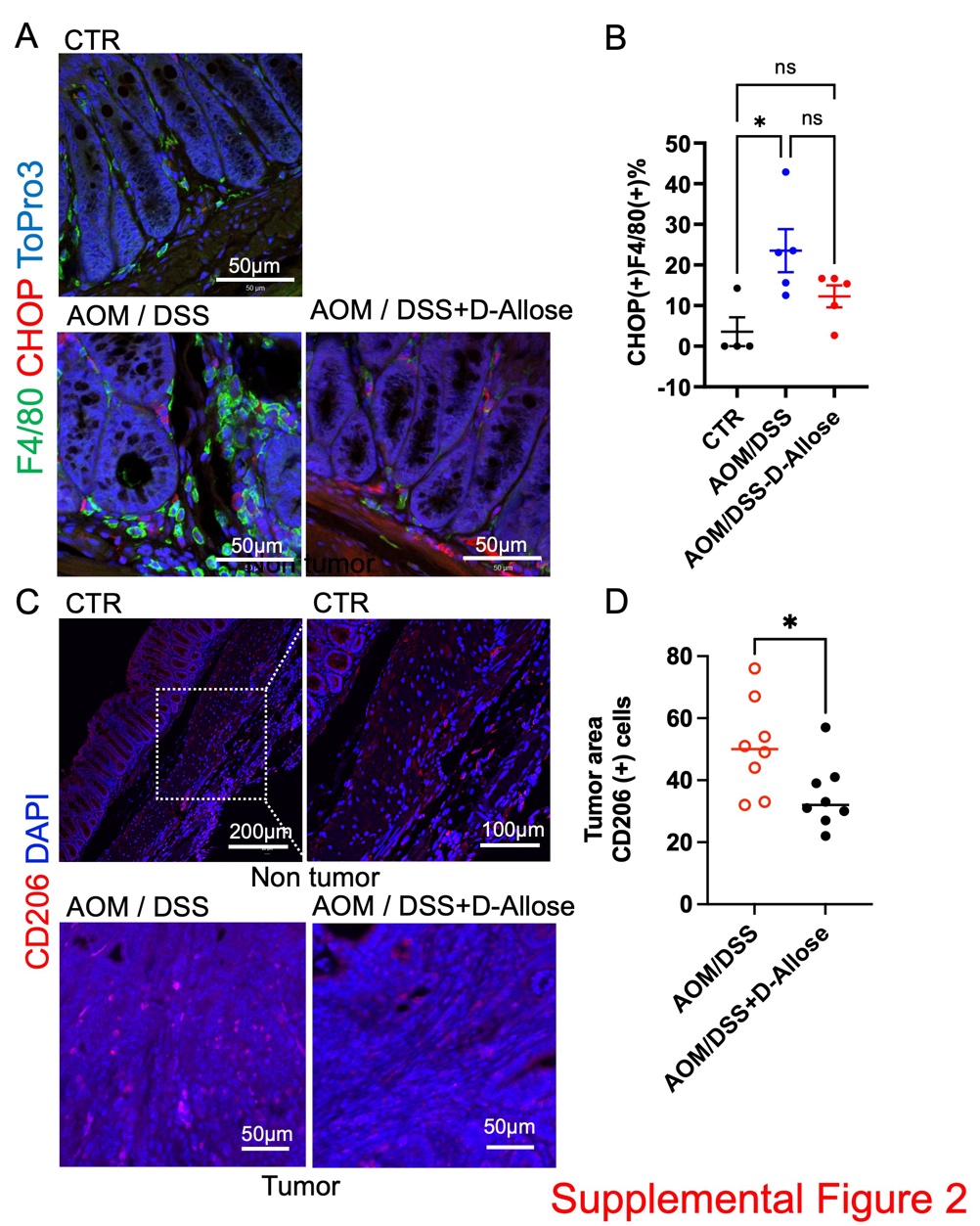


A, B: Co-staining of F4/80 and CHOP in non-tumor area of mice colon. C, D: Immunostaining of CD206 in the normal area of control mice and tumor area of AOM / DSS, AOM / DSS + D-allose mice. Positive cell numbers were normalized to ToPro3 or DAPI-positive nuclei (n = 5). ns p＞0.05, * p < 0.05

**Supplementary Figure 3. ER stress evaluation in RAW264.7 cells**


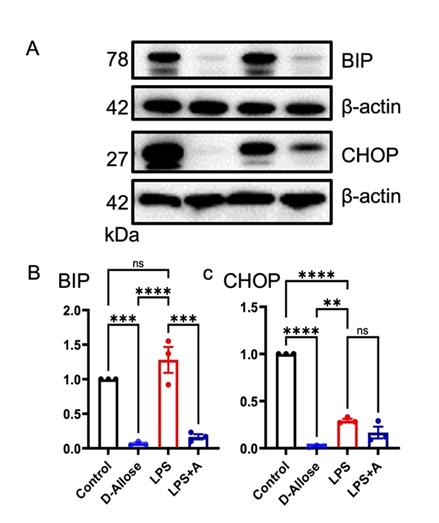


A, B: Western blot analysis of BiP and CHOP in RAW264.7 cells treated with or without LPS (1 μg/mL) or D-allose (10 or 50 mM) for 24 hours (n = 4).

**Supplementary Figure 4. TXNIP siRNA silencing efficacy, Glut1 expression, and pMLC2 localization in Caco-2 cells**


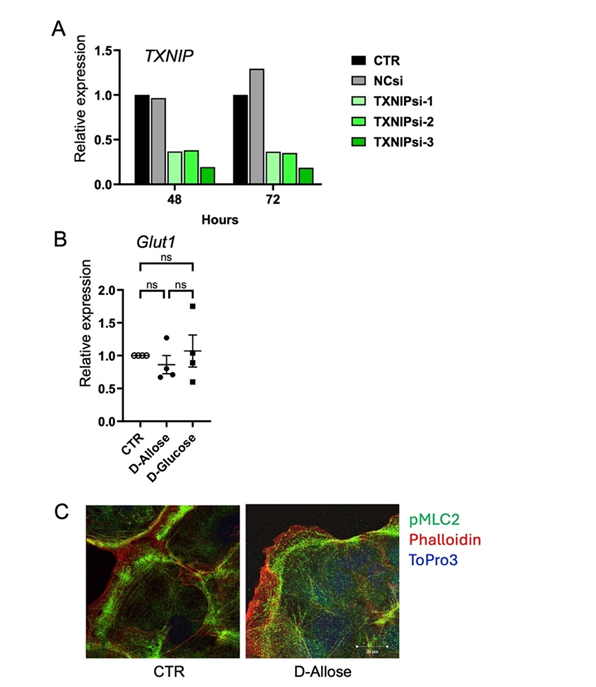


A: qPCR analysis of the knockdown efficiency of three TXNIP-targeting siRNAs (TXNIPsi-1, TXNIPsi-2, and TXNIPsi-3) and negative control siRNA (NCsi) in cultured Caco-2 cells (n = 4). B: Glut1 mRNA expression in Caco-2 cells treated with D-allose or D-glucose (n = 4). ns p＞0.05. C: Immunofluorescence staining for pMLC2 and phalloidin (actin, red) in Caco-2 cells treated with or without D-allose (50 mM) for 24 hours.

**Supplementary Figure 5. Effect of D-allose on mitochondrial respiration of Caco-2 cells**


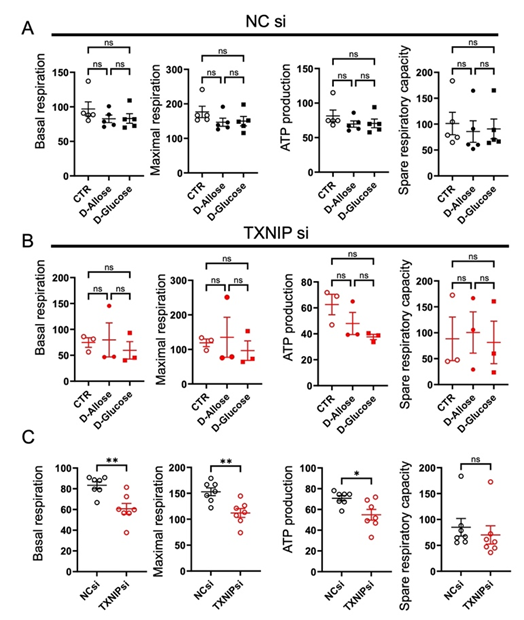


A: Mitochondrial respiration in Caco-2 cells transfected with negative control siRNA (NCsi) and treated with 50 mM D-allose or D-glucose for 72 hours (n = 4). B: Mitochondrial respiration of Caco-2 cells transfected with TXNIP-targeted siRNA (TXNIPsi) and treated with 50 mM D-allose or D-glucose for 72 hours (n = 4). C: Comparison of mitochondrial respiration between NCsi- and TXNIPsi-transfected Caco-2 cells. ns p ＞0.05 * p < 0.05, ** p < 0.01

# Supplementary Raw Data

Supplementary Data 1. Western blot raw data corresponding to Figure 3D.


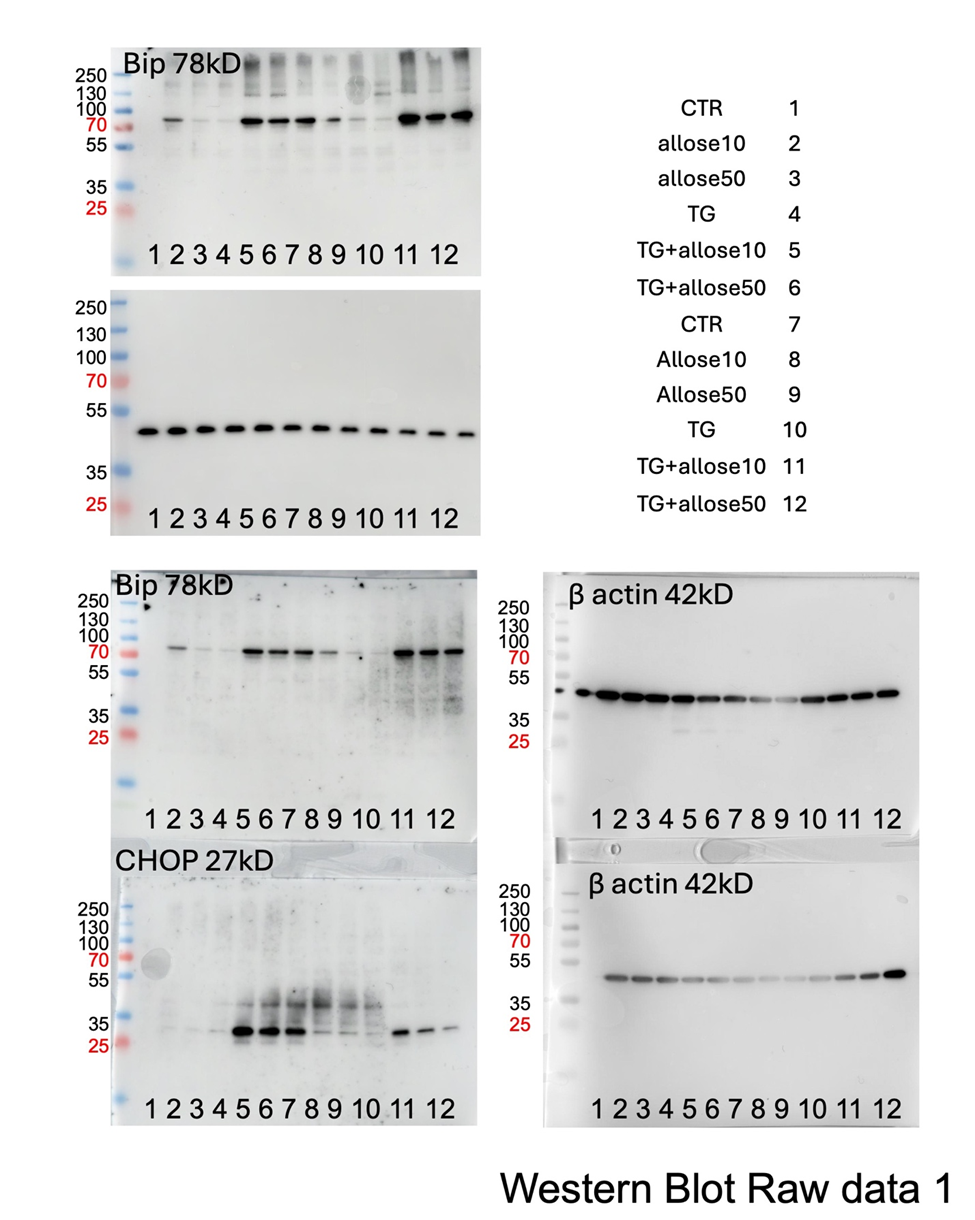


Supplementary Data 2. Western blot raw data corresponding to Supplementary Figure 1.


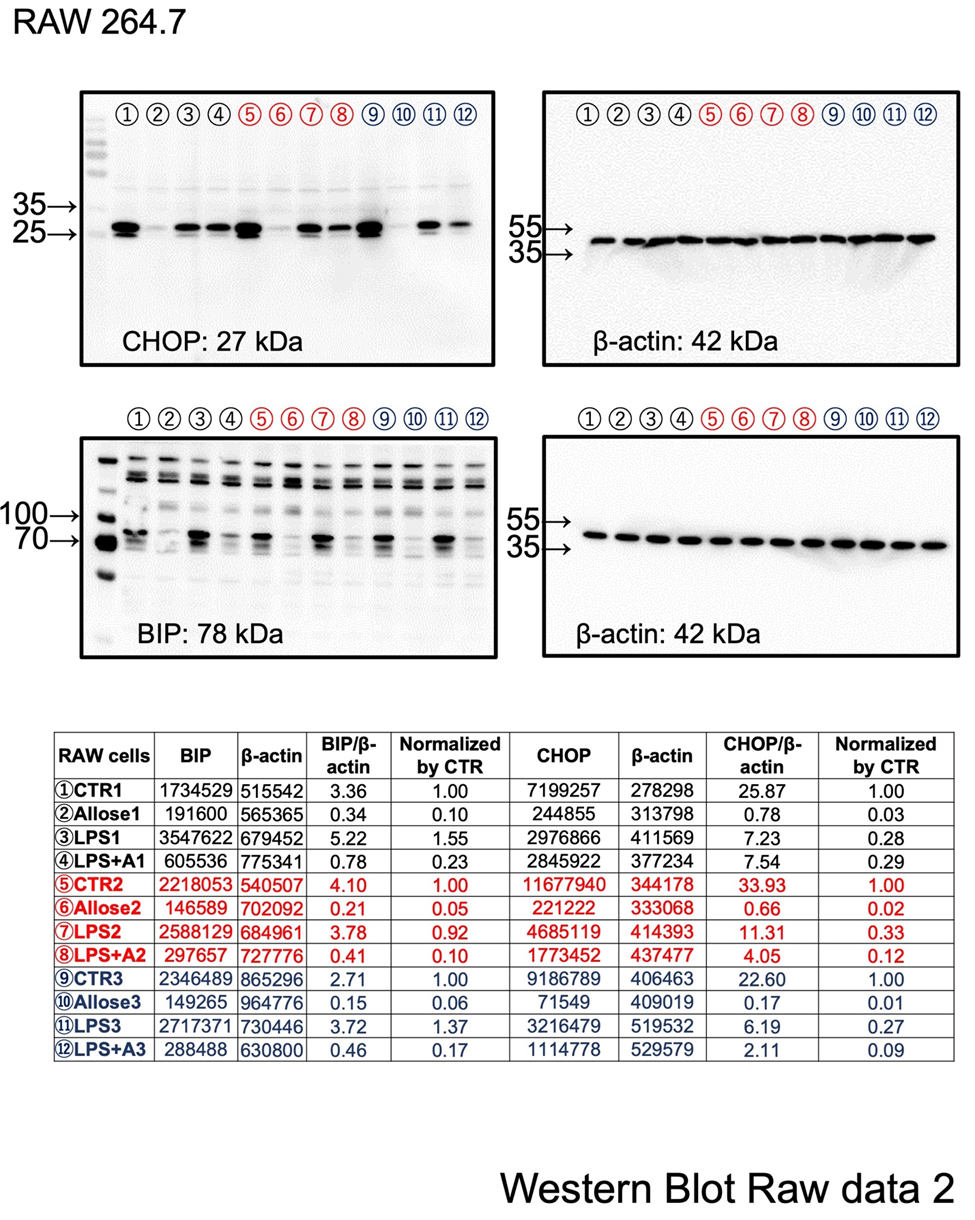


Supplementary Data 3. Western blot raw data corresponding to Figure 7F.


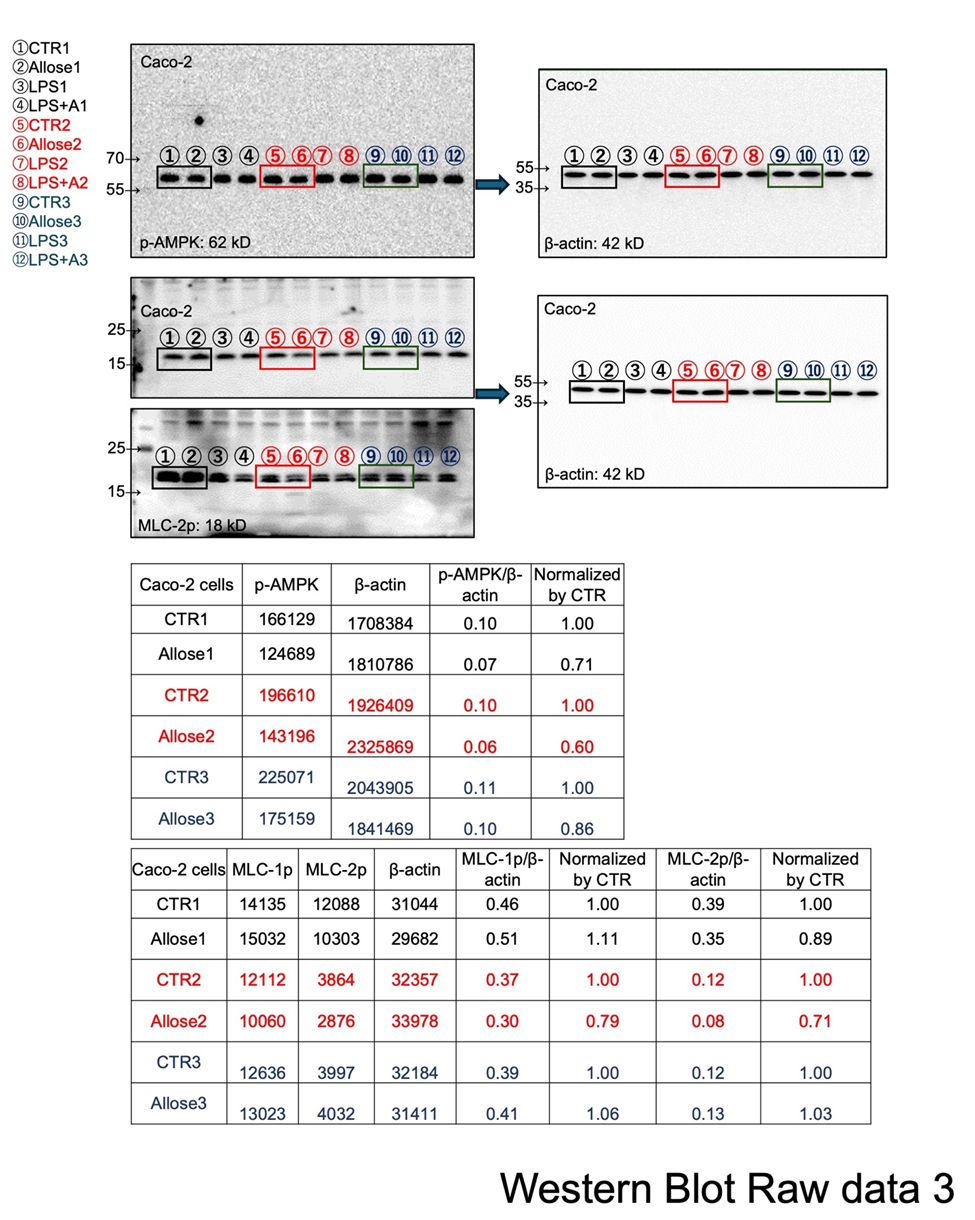

Supplement: Supplementary file 1 [file DataSheet1.docx]
